# Discovery of a Small-Molecule Modulator of the Autophagy Lysosome Pathway That Targets Lamin A/C and LAMP1, Induces Autophagic Flux, and Affects Lysosome Positioning in Neurons

**DOI:** 10.51894/001c.164011

**Published:** 2026-07-31

**Authors:** Sruchi Patel, Amanda Snead, Swetha Gowrishankar

**Affiliations:** 1 College of Osteopathic Medicine, Michigan State University

## 11

### Introduction

Impairment of the autophagy–lysosome pathway that help turn over proteins and organelles in neurons, contributes to pathology of neurodegenerative diseases. Identifying small molecules that enhance autophagic flux and modulate lysosome function in neurons are thus of interest, as they have therapeutic potential for such disorders.

### Objectives

Determining effect of the small molecule RH1115 on lysosomal positioning in neurons, as well as its effect on cell survival.

### Methodology

Human iPSC-derived i^3Neurons were treated with RH1115 and DS1040 and its structural analogs. Neuronal viability, lysosome localization, and vesicle morphology were assessed by high-resolution confocal microscopy. Lysosome position within cell bodies was evaluated in a blinded fashion, LAMP1 expression and vesicle intensity were quantified by immunostaining and image analysis.

### Results

Treatment of i^3Neurons with DS1040, RH1096, RH1103, and RH1115 significantly altered lysosome positioning, inducing enhanced perinuclear clustering of LAMP1-positive vesicles. Notably, RH1115 increased the intensity and mean size of LAMP1 vesicles, consistent with elevated lysosomal protein content.Together with data from other experiments in this study, RH1115 was validated as a dual modulator of Lamin A/C and LAMP1 that promotes autophagic flux while remodeling lysosome positioning in neurons.

### Conclusion

We show that RH1115, a novel small-molecule modulator of the autophagy increases expression of key lysosomal protein, LAMP1, and mobilizes lysosomes inward /towards nuclear region. This in turn, could be linked to the induction of autophagy. Future studies will determine if the two effects of the small molecule are linked.

